# Massive structural and compositional changes over two decades in forest fragments near Kampala, Uganda

**DOI:** 10.1002/ece3.747

**Published:** 2013-09-12

**Authors:** C Bulafu, D Baranga, P Mucunguzi, R J Telford, V Vandvik

**Affiliations:** 1 Department of Biology, School of Natural Sciences, Makerere UniversityP. O. Box 7062, Kampala, Uganda; 2 Department of Biology, University of BergenThormøhlensgate 53A, N-5006, Bergen, Norway

**Keywords:** Biodiversity, conservation, ownership, REDD, red-listed species, resampling, turnover

## Abstract

Private forests harbor considerable biodiversity, however, they are under greater threat than reserved areas, particularly from urbanization, agriculture, and intense exploitation for timber and fuel wood. The extent to which they may act as habitats for biodiversity and how level of protection impacts trends in biodiversity and forest structure over time remain underresearched. We contribute to filling this research gap by resampling a unique data set, a detailed survey from 1990 of 22 forests fragments of different ownership status and level of protection near Kampala, Uganda. Eleven of the 22 fragments were lost over 20 years, and six of the remnants reduced in size. Forest structure and composition also showed dramatic changes, with six of the remnant fragments showing high temporal species turnover. Species richness increased in four of the remaining forests over the resample period. Forest ownership affected the fate of the forests, with higher loss in privately owned forests. Our study demonstrates that ownership affects the fate of forest fragments, with private forests having both higher rates of area loss, and of structural and compositional change within the remaining fragments. Still, the private forests contribute to the total forest area, and they harbor biodiversity including IUCN “vulnerable” and “endangered” species. This indicates the conservation value of the fragments and suggests that they should be taken into account in forest conservation and restoration.

## Introduction

Human-induced land-cover conversion is one of the primary determinants of environmental change and a major threat to biodiversity, ecosystem services, and function (Millennium Ecosystem Assessment 2005; Schleuning et al. 2011). Tropical forest loss is estimated at 58,000 km^2^ annually and projected to increase, leading to habitat loss, fragmentation, and species extinctions (Laurance et al. 2009; Wright 1990). Approximately 90% of the remaining tropical forest biome is found outside reserved areas (Chape et al. 2003), as remnant fragments of natural vegetation embedded in landscapes are devoted primarily to human activities. They are facing intense competition from alternative land uses and as such there is an urgent need to increase conservation efforts both in reserved and private forests to reduce biodiversity loss (Millennium Ecosystem Assessment 2005; Schmitt et al. 2008; CBD 2010) as well as to reduce emissions from deforestation and degradation (REDD+). Private forest fragments owned by individuals, families, organizations, tribes, or the forestry industry are becoming central in this new conservation paradigm (Liu et al. 2007).

Private urban forests harbor considerable biodiversity and provide an array of goods and ecosystem services including soil stabilization, erosion control, pollutant filtering, as well as carbon storage (Millennium Ecosystem Assessment 2005; FAO 2011), and provide habitats for native, threatened and/or endangered species (Barlow et al. 2007; Edwards et al. 2011). However, they are under greater threat than protected areas, particularly from agriculture, urbanization, and intense exploitation for timber and fuel wood (Webb 2002; Naughton-Treves et al. 2005; Chatterjea 2012). Yet despite their crucial role, the extent to which they act as habitats for biodiversity remains underresearched. Although long-term effects of land use and land-use change on intact tropical forest structure and composition has been well studied using permanent plots of mostly ≤1 ha in size (Sukumar et al. 1992; Zimmerman et al. 1994; Condit et al. 1996; Lwanga et al. 2000; Taylor et al. 2008), private forests and forest fragments in urban and semiurban environments have not been featured strongly in this body of work particularly in tropical Africa. Even the few that have been studied have not benefited from the existence of long-term permanent plots and hence we cannot compare dynamics and rates of change in forests under different ownership. We do not know how much they change relative to forest reserves in terms of forest structure, species richness, and species composition, given their lower protection status. In lieu of long-term monitoring data, resampling of historical data offers opportunities to assess trends (Sheil et al. 2000; Taylor et al. 2008) and effects of different management practices (Norden et al. 2009) and regulatory regimes.

In Uganda, 30% of the tropical forests are reserved and 70% are on private land (National Environmental Management Authority 2007). From 1990 to 2005, an estimated 1.3 million hectares of private forest (62% of the total private forest area) was lost to agriculture and urbanization (National Forestry Authority 2008; Obua et al. 2010). This is almost double the rate of deforestation found in the reserved forests (Obua et al. 2010). For example, the once extensive forests of the Kampala area have been reduced and fragmented into small forests restricted to lake shores, valleys, and hilltops (Baranga 2004a,b) surrounded by urban, semiurban areas, and subsistence agricultural gardens. Forest owners include the Catholic Church, private landowners, government institutions, and community ownership for cultural use (sacred forests) with associated management patterns. Ownership affects access rights and exploitation intensity.

We predict that differences in forest management associated with different ownership will lead to greater risk of forest loss and more structural and compositional change in private forests with low levels of protection, and least change in government-owned, particularly reserved, forest. To test these predictions, we resampled a data set from 1990 of 22 forest fragments whose ownership ranged from reserve, research, and sacred to individually owned forests (Baranga 2004a,b). We use plot-based methods to examine forest structure and tree species compositional changes within and among forest fragments. This allows us to compare species composition and structural change in forest fragments over a 20-year period in the same landscape, but differing in ownership and level of protection.

## Material and Methods

### Study area

**Figure 1:**
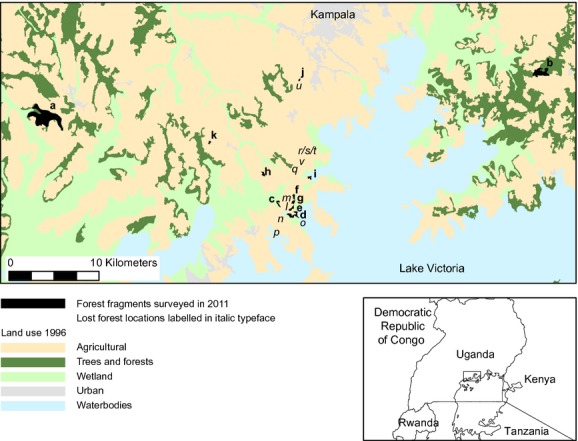
Map showing location of the study area and forest fragments. Full names for sites a-k can be found in Table 1.

The study was carried out in the greater Kampala area in central Uganda (0°05′N–0°16′N and 32°30′E–32°38′E; at altitudes 1200–1250 m.a.s.l; Fig. 1). The area encompasses medium-altitude moist evergreen forests of type C2 *Piptadeniastrum–Albizia–Celtis* forest (Langdale-Brown et al. 1964). The region experiences bimodal rainfall, with an annual rainfall of 1350 mm and a mean temperature of 25°C (National Environmental Management Authority 2007). The topography varies from flat areas to low hills.

### Study sites

In 1990, baseline data on species composition and structure of 22 forest fragments in the greater Kampala area were collected by Baranga (2004a,b). We aimed to resample these forests 20 years after the original survey. Eleven of the forests could not be resampled, because they had been cleared; and the remaining eight private and three government-owned forests (a, b, and c) were resampled as described below. Forest (a) is a reserved forest managed by National Forestry Authority located inland and was the nearest reserve to the fragments sampled in this study and was included for comparative purposes. It is a medium-altitude moist evergreen forest about 4.5 km^2^ of type C2 *Piptadeniastrum–Albizia–Celtis* forest (Dawkins and Philip 1962). Forest (b) is also located inland and has cocoa and tea experimental plots regenerating naturally after they were abandoned 50 years ago and is managed by Coffee Research Institute. Forest (c) is located near the shores of Lake Victoria and is managed by Uganda Virus Institute (for a description of this site, see Eggeling 1940). The private forests are on land managed by private landowners, although forest products are harvested with guidance from the district forest officer and in accordance with the management plans and regulation made under the Forest and Tree Planting Act (2010), although this is hardly implemented. They included four church-owned forests (d, e, f, and g); three individually owned (h, i, and j); and one sacred forest controlled and managed by a local community (k) where one of the Buganda clans perform cultural rituals related to power and healing. The lost forests (l–u) are shown in Figure 1 and listed in Table 1. Six of the private forest fragments, four extant (d, e, g, and i) and two lost (n and o), were part of the once extensive lake shore forest of Mengo district situated on the Entebbe Peninsula covering the short slopes adjacent to the papyrus swamps around Lake Victoria and less than 3 km apart (Table 1). The church forests (d–g, l, and n) were once part of a continuous forest block along the shoreline adjoined by a large expanse of papyrus swamp on the eastern side. Forest (d) is separated from the former by open scrubland along the lake shore and is bordered by a small swamp on the western arm and a vast papyrus swamp on the south side. The church forests are bordered on the east side by low population density homesteads interspersed with scrub and scattered large trees. Forests (h, j, k, t, and u) are, or were, located inland, sometimes on hilltops and rocky soils in a landscape that was dominated by low population density settlements interspersed with large trees and scrub. Ownership type did not change between the two surveys for any of the fragments.

**Table 1:** Summary of changes in forest fragments in Kampala area for the period from 1990 to 2010

			Number of transects		Number of plots		Forest size (ha)		Forest status		Population density	Level of protection	Mean gap fraction	Forest location	Ownership
											
Forest name	Forest code	Location	1990	2010	1990	2010	1990	2010	1990	2010	2010				
Mpanga	a	0°13′N, 32°18′E	5	5	50	48	476	438	1	1	2.25	Strict	0.68	Inland	Government
Kituza	b	0°16′N, 32°47′E	5	5	48	49	110	110	1	1	0.00	Strict	0.55	Inland	Government
Zika	c	0°07′N, 32°31′E	3	3	25	23	13	13	1	1	8.00	Strict	0.75	Shoreline	Government
Kibale	d	0°07′N, 32°32′E	4	4	27	31	42	38	2	2	0.75	Weak	0.79	Shoreline	Church
Kisubi Technical	e	0°07′N, 32°32′E	4	4	20	18	16	10	2	2	3.50	Weak	0.76	Shoreline	Church
Gogonya	f	0°07′N, 32°32′E	4	4	21	21	15	11	2	3	4.00	Weak	0.72	Shoreline	Church
Kisubi Girls	g	0°07′N, 32°32′E	5	5	47	18	15	13	3	3	0.25	Weak	0.83	Shoreline	Church
Wamala	h	0°09′N, 32°31′E	6	6	31	30	19	19	2	2	2.25	Weak	0.63	Riverine	Individual
Nzuki	i	0°09′N, 32°33′E	5	5	20	17	10	10	2	2	1.25	Strict	0.70	Shoreline	Individual
Bunamwaya	j	0°15′N, 32°33′E	7	7	26	23	9	9	3	2	33.75	Strict	0.76	Hill top	Individual
Katwe	k	0°11′N, 32°27′E	4	4	42	18	11	5	2	2	9.00	Strict	0.83	Hill top	Sacred
Kisubi paddock	l	0°07′N, 32°32′E					1	0	3	SE	3.50		–	Shoreline	Church
Kisubi hospital	m	0°07′N, 32°32′E					2	0	3	PF	4.25		–	Shoreline	Church
Kanywa	n	0°07′N, 32°32′E					9	0	2	SE	27.00		–	Shoreline	Individual
Nabinonya	o	0°07′N, 32°32′E					8	0	3	T	3.70		–	Shoreline	Individual
Nalugala	p	0°09′N, 32°33′E					16	0	2	SE	28.00		–	Shoreline	Individual
Namulanda	q	0°09′N, 32°33′E					12	0	4	SE	1.50		–	Shoreline	Individual
Nganjo A	r	0°10′N, 32°33′E					4	0	4	SE	1.50		–	Shoreline	Individual
Nganjo B	s	0°10′N, 32°33′E					4	0	3	SE	7.25		–	Shoreline	Individual
Nganjo C	t	0°10′N, 32°33′E					4	0	2	SE	8.50		–	Shoreline	Individual
Seguku	u	0°14′N, 32°33′E					13	0	4	SE	17.50		–	Riverine	Individual
Mawanyi	v	0°10′N, 32°33′E					12	0	2	HG	89.50		–	Riverine	Individual

Forest areas and extent are based on LANDSAT image from 27 February 1989 and aerial photography from 2010. Level of protection was based on whether or not there were full time foot patrols by a forest guard. Gap fraction is only available for forests in the 2010 survey. Forest status: 1, fairly intact; 2, disturbed; 3, degraded; 4, highly degraded. SE, lost to settlement; T, lost to tourism development; PF, lost plantation forestry; and HG, lost to home gardens.

### Field methods

The method used in this study was designed by Baranga in 1990 (Baranga 2004a,b) and this was followed as closely as possible in 2010. For each forest fragment line, transects ranging in length from 100 m to 1000 m, depending on forest size, were established along the longest axis of each forest. In forest (a), transects were established along the already established trail system but only in the dry parts of the forest. Neighboring transects were 30 m apart. Transects started 10 m from the edge of the fragment. Plots of 10 × 5 m were established at 50-m intervals along the transects. Within each plot, the diameter at breast height (DBH) of each tree (woody individuals with DBH ≥ 3.7 cm) was measured at 1.3 m. DBH of species with buttresses higher than the breast height was measured above the buttress whenever possible and is referred to as the diameter at reference height (DRH) (Sheil et al. 2000). Basal area ha^−1^ for each species was calculated. Nomenclature followed Eggeling and Dale (1951) and Hamilton (1981). Voucher specimens are deposited at Makerere University Herbarium. Palms were not enumerated in the 1990 survey so the few palms encountered in this study were omitted. The number of transects established and plots sampled are indicated in Table 1.

The disturbance status of the forest fragments was assessed by allocating scores from 1 to 4 (1 – fairly intact; 2 – disturbed; 3 – degraded, 4 – highly degraded). This was based on the following criteria: (1) presence of a distinct upper, middle canopy, ground layer, low density of undergrowth; (2) presence of a distinct upper, middle canopy, ground layer, high density of undergrowth; (3) presence of distinct upper and middle canopy with high number of gaps and evidence of human activities such as tree cutting and forest clearing; and (4) presence of only one distinct upper canopy, no or low undergrowth, and high number of tree cutting. Land-use change for lost forests and forest ownership was documented. Level of protection was categorized as strict if a guard was employed or weak if no guard was employed by the forest owners. We quantified housing density for 2010 sample regime as an estimate for population from Google Earth maps by estimating the mean number of houses in four rectangular plots measuring 0.5 km × 0.1 km at the edge of the forest in north, east, west, and south directions (Google Earth 6.1 2011). Gap fraction, the fraction of view looking up from beneath the canopy that is not blocked by wood and foliage, was estimated using hemispherical photography. Photographs were acquired with a skyward facing camera (Nikon D60; Nikon Inc., Melville, NY) equipped with Tonika fish-eye lens oriented to magnetic north placed at the center of each 10 × 5 m plot, 70 cm above the ground. One photograph was taken per plot, in jpeg format at a resolution of 2592 × 3872 pixels. An automatic binary classification (Option 1: nonselected pixels were considered as vegetation) was performed to compute the gap fraction P0(57.5°) using CAN_EYE software (version 5.0) (Demarez et al. 2008, http://www.avignon.inra.fr/can_eye). A gap fraction of zero means the sky is completely obscured in the particular sky sector, whereas a gap fraction of one means the sky is completely visible. Forest boundaries in 2010 were delineated from aerial photographs. The resulting shapes were compared to 2010 and 1990 LANDSAT images (30-m resolution TM composites) in Esri ArcGIS 10.0 (ESRI, Redlands, CA), to outline the 1990 boundaries and calculate forest area.

### Data analysis

Forest lost over time was assessed by visiting areas surveyed in the baseline study (Baranga 2004a,b). Forest area loss was calculated as the difference in area over the two time periods. To assess forest structural change, we compared mean stem counts per hectare of each forest over time. This was carried out separately for the following different size classes: >40; 20–40; 10–20; and <10 cm DBH.

To compare species richness among sites and sampling years, individual-based rarefaction curve (bootstrap resampling) with 95% confidence intervals was used. This controls for the effect of differences in sampling area or density between sites or sampling years. We used 500 randomizations for the bootstrap resampling. Sampling with replacement was preferred over sampling without replacement because it leads to a variance among randomizations that is meaningful. The bootstrap resampling was performed using EstimateS (Colwell 2006).

We assess forest compositional change over space and time in two ways. First, we assess the overall pattern in species composition within and among forests using nonmetric multidimensional scaling (NMDS; Faith et al. (1987) on Bray–Curtis distances of the species relative abundances in each forest fragment. Second, we estimated the taxonomic turnover of each forest over time in relation to overall compositional patterns in the data set using the vegdist function with Bray–Curtis dissimilarity index in the vegan library (version 2.0-6.) (Oksanen et al. 2012). For each forest, we first estimated the taxonomic distance between the 2010 and 1990 vegetation and then, for comparison, we assessed the taxonomic distance for each of the 2010 forests with the 1990 and 2010 landscapes. To explore compositional changes over time in different size classes, we performed Bray–Curtis dissimilarity analyses for data subsets consisting of the following DBH size classes: ≤10 cm, 10–20 cm, 20–40 cm, and >40 cm. With the exception of rarefaction, all the above analyses were performed on relative abundance data on species with the abundance of more than two in order to control for variation in sampling effort. All analyses were done using the R statistical language version 2.0-6 (R Core Team 2012) with the vegan library (R version 2.0-6) (Oksanen et al. 2012).

We tested whether site configurations in NMDS plots were related to forest size, disturbance index, level of protection, and forest location, and by testing their significance with a permutation test (1000 iterations). To investigate the effects of level of protection, disturbance index, and human population density on forest loss, stem counts, rarefied species richness, and turnover, we used nonparametric Kendall's rank correlation tau test while Kruskal–Wallis rank sum test was used to test the effect of ownership and location on all response variables considered above.

## Results

### Changes in forest structure

Of the 22 forest fragments surveyed in 1990, only 11 remained after 20 years. All the forests lost were privately owned. We found that forests with higher disturbance in 1990 and forests in areas with a population density were more likely to be lost (Tables 1 and 2). Six of the remaining forests became smaller, with loss ranging from 7% to 54% of the total area with the rest showing no change in size over the resample period (Table 1). In 2010, the gap fraction, a measure of canopy openness, and amount of light penetrating to the forest floor ranged from 0.55 to 0.83. We found that gap fraction decreased with an increase in forest size (Kendall's tau = −0.45, *P* = 0.1).

**Table 2:** Effects of level, protection, disturbance index, human population density, ownership, and location on forest loss, stem counts, rarefied species richness, and taxonomic turnover, and for the different DBH (cm) size classes in Kampala forest fragments, Uganda

	Response variables											
	
		Stem counts/ha						Turnover				
						
Predictor variables	Forest loss	<10 cm	10–20 cm	20–40 cm	>40 cm	Species richness	Total turnover	<10 cm	10–20 cm	20–40 cm	>40 cm	NMDS *r*^2^
Level of protection	0.196	0.510	0.662*	0.459	0.255	−0.510	−0.612*	0.104	0.204	0.510	0.309	0.304*
Disturbance index	0.652**	−0.674**	−0.629**	−0.450	−0.270	−0.629*	0.719**	0.206	0.135	−0.180	−0.068	0.495**
Population density	0.372*	0.293	0.110	−0.330	0.220	0.294	−0.056	−0.075	−0.110	−0.073	−0.093	0.44*
Ownership	14.50**	7.000	8.599	6.591	7.462	7.136	8.258	2.833	2.318	4.712	4.037	0.628*
Location	10.33*	4.606	1.773	3.046	2.909	4.561	3.242	5.428	2.864	2.500	0.643	0.518*

Given are the Kendall's tau rank correlations for level of protection, disturbance, and population density, whereas Kruskal–Wallis chi-square is given for ownership and location except for forest loss as response variable where we used chi-square test. All predictor variables are based on 2010 estimates except when forest loss was taken as a response variable, in which case the predictor variables were based on both 1990 and 2010 estimates. All response variables are based on proportional changes rather than on absolute value except turnover and forest loss. *N* = 11 for all response variables except forest loss where *N* = 22, and degrees of freedom for ownership and location were 4 and 3, respectively. Also included in the last column are the goodness-of-fit analyses from NMDS for the five predictor variables. Variable type: level of protection=binary, disturbance=rank, population density=numerical, ownership=categorical, location=categorical, forest loss=binary, and stem counts and turnover=numerical.

*P*-values are indicated as stars next to the Kendall's tau and χ^2^, where *P = 0 0.05 and **P = 0.01.

**Figure 2:**
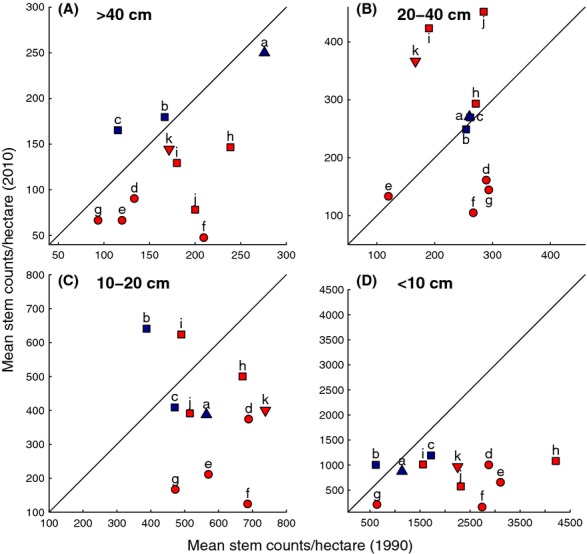
Size class changes (mean stem counts per hectare) over the resample period in (A) >40 cm DBH, (B) 20–40 cm, (C) 10–20 cm, and (D) <10 cm DBH in forest fragments (a–k). Full names for sites a-k can be found in Table 1. Black triangle: protected forest; black squares: research forests; grey squares: individually owned forests; grey circles: church forests; and grey triangle: sacred forest. The diagonal line is 1:1 line.

Over the resample period, reserved (a) and research forests (b and c), all government owned with strict access control and low disturbance levels, showed low changes in mean stem counts per hectare in large size classes (>40 cm DBH) and small size class <10 cm DBH (Fig. 2). Church forests (forests d–g) which had no or weak access control show large decreases in stem counts over the resample period, particularly for small- (<10 cm DBH) and midsized trees (10–20 cm DBH) (Fig. 2). We found that small and medium stem counts loss was higher with higher disturbance index (Table 2). There were dramatic increases in the densities of stems 20–40 cm DBH forests (k, i, and j) as well as forest (b) in the ≤10 cm DBH (Fig. 2B and D). The sacred forest (k) showed variable changes across size classes (Fig. 2A).

### Species richness and community composition over time

**Figure 3:**
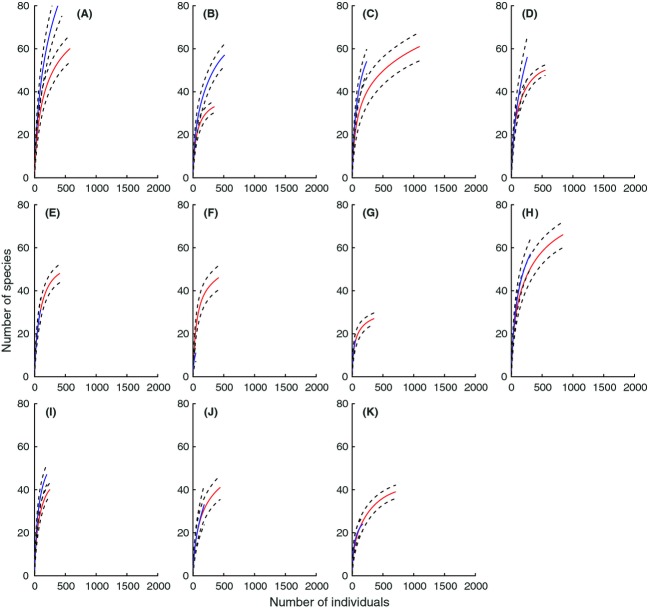
Individual-based rarefaction curves for 2010 (blue line) and 1990 (red line) for each of the forest fragments. Dashed lines are 95% confidence intervals. Forest names can be found in Table 1. * [Correction added on 17 September 2013, after first online publication: this figure has been corrected to show the correct rarefaction curves.]

Species richness, as reflected in the rarefaction curves, varied among forest fragments and sample periods (Fig. 3). The mature reserved forest had the highest species richness (Fig. 3A) and heavily disturbed forests had the lowest species richness in both sample periods (Table 1; Fig. 3F and G). We found that rarefied species richness decreased with an increase in disturbance (Table 2). Between the two surveys, species richness increased in four forests (Fig. 3A–C, and I) and decreased in two (Fig. 3E and F). Five forests showed minimal changes (Fig 3D, G, H, J, and K).

**Figure 4:**
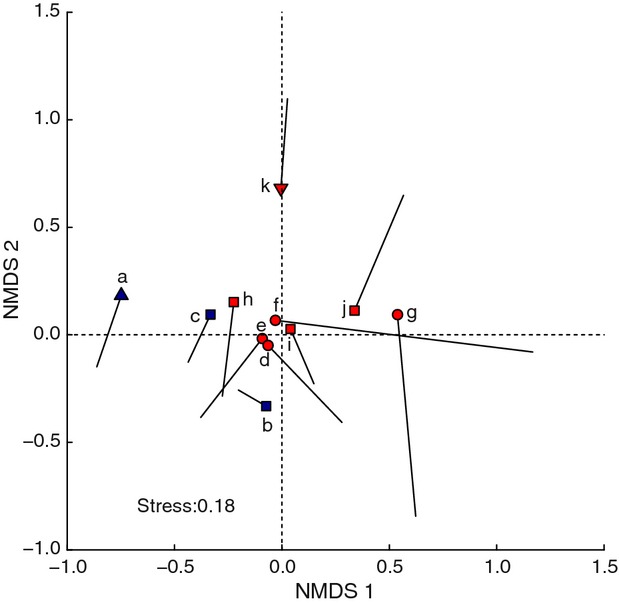
Non-metric dimensional scaling (NMDS) plot showing response of tree community over 20-year period for trees ≥3.7 cm DBH in 11 forest fragments in and around “Kampala area,” Uganda. Long line length indicates large changes and short line length shows minimal changes over the resample period. The labeled end points for the lines are 2010 positions. Forest names can be found in Table 1. For symbols, see Figure 2. * [Correction added on 17 September 2013, after first online publication: the labels on the points are added for this figure.]

The NMDS reflects compositional differences in tree communities across forest ownership and disturbance gradient, with the reserved intact forest on the far left of the first axis and degraded forest to the far right, and disturbed ones in the center (Fig. 4). Fragments varied in species composition over space and time, but there was no consistent trend over the 20 years between surveys, as reflected by the large variation in the length and direction of the arrows adjoining the 1990 and 2010 position of each forest in the NMDS diagram, although species composition of the church forests seemed to be similar in later sampling (Fig. 4). The permutation test showed that disturbance index, protection level, location, and ownership were significant variables in the ordination (Table 2).

**Figure 5:**
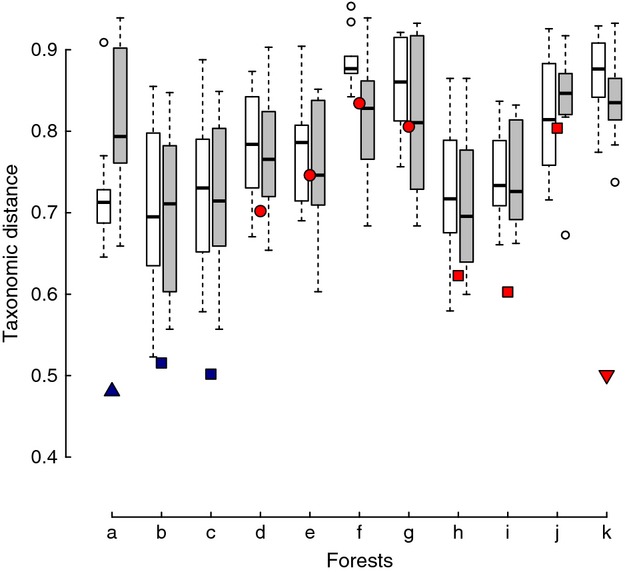
Bray–Curtis dissimilarity measures for all stems combined (>3.7 cm DBH) for forest fragments in and around “Kampala area,” Uganda, in 1990 and 2010. The symbols represent the taxonomic turnover for each fragment; the box plots show the differences in the target fragment from all fragments in 1990 (white) and 2010 (grey). Forest names can be found in Table 1. For symbols, see Figure 2.

The forests show a wide range in compositional turnover (Bray–Curtis dissimilarities 0.48–0.83). Reserved, research, and sacred forests (a, b, c, and k) show relatively low taxonomic turnover, that is, they have stable species composition over the 20-year period (Fig. 5, dark blue symbols and red triangle), whereas the majority of church and individual forests (d–j) have relatively high species turnover with time (Fig. 5, red circles and squares). We found that species turnover was low when disturbance index was low and level of protection was high (Table 2). These compositional changes over time are also reflected at landscape level. The reserved forest with the lowest species turnover was more similar to the 1990 landscape (Fig 5 a white box plot), whereas research and two individually owned forests show minimal differences in similarity to the forests in the landscape (Fig. 5 b–c, h, and i, white and gray box plots). Forests with high species turnover tended to be more similar to current landscapes (Fig. 5 d–k, gray box plots). Church forest (f) changed dramatically in species composition and it is more similar to modern landscape (Fig. 5 f, gray box plots), as seen from it's extremely high taxonomic turnover, and is also very different from any other forests in the landscape.

### Compositional changes across size classes

**Figure 6:**
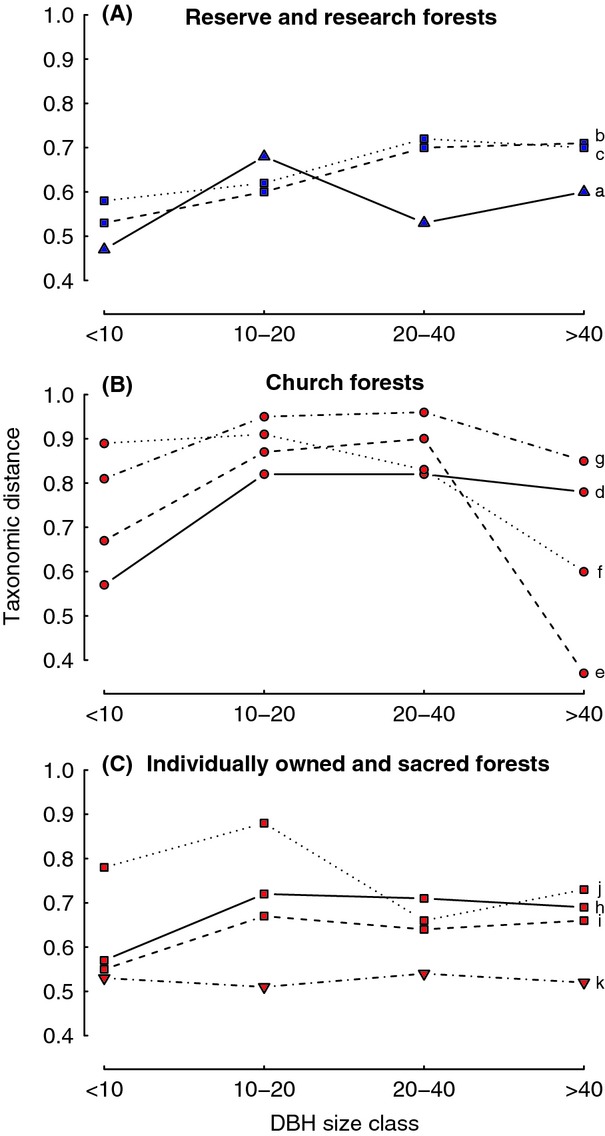
Turnover (Bray–Curtis dissimilarity) for different size classes of tree stems for (A) reserve and research forests, (B) church forests, and (C) individually owned forests and sacred forests near Kampala area, Uganda. Forest names can be found in Table 1. For symbols, see Figure 2.

In reserved and research forests, there was low compositional turnover across all size classes (Fig. 6A). In contrast, church forests had high compositional turnover in the smaller size classes, increasing among the young trees (10–20 and 20–40 cm size classes DBH), and relatively low compositional turnover in the largest size classes (>40 cm DBH) with forest showing least species turnover in the largest size class (Fig. 6B). Among individually owned and sacred forests, compositional turnover was generally moderately high across size classes with the sacred forest having a lower turnover than the reserved and research forests, and with forest (j) having the highest compositional turn over in the small and young tree classes (Fig. 6C).

There was a general trend for subcanopy pioneer species characteristic of wetter environments such as *Musanga cercropioides* R. Brrr & Tedlie that dominated in 1990 to be replaced by light demanders that thrive on relatively dry areas (*Pycnanthus angolensis* [Welw.] Warb., *Antiaris toxicaria* [Rumph.ex Pers.] Lesch., and *Maesopsis eminii* Engl. [Appendix 1]). In the 2010 census, one invasive species, *Senna spectabilis* (DC.) Irwin & Barneby, was recorded in forest (j), an individually owned forest, at low density. The exotic fruit tree *Artocarpus heterophyllus* Lam. was found in eight of the forest fragments in 2010 compared to three in 1990. Species categorized by the IUCN red list (IUCN 2011) as vulnerable and near threatened are found across all ownership types with minimal changes in species numbers over time (Table 3).

**Table 3:** IUCN species numbers and total abundance in parenthesis for vulnerable and near-endangered categories in the different forest ownership types for the two sample periods

Forest ownership type	1990		2010	
		
	Saplings	Trees	Saplings	Trees
Reserve	3 (9)	3 (6)	4 (10)	4 (7)
Research	3 (15)	4 (16)	3 (39)	1 (5)
Church	2 (8)	3 (8)	3 (15)	3 (4)
Individual	3 (12)	1 (5)	3 (26)	2 (2)
Sacred	1 (24)	1 (14)	1 (14)	1 (10)

Tree >10 cm DBH and saplings <10 cm DBH.

## Discussion

Our results show dramatic changes in forest structure and composition of forest fragments in the greater Kampala area over a 20-year period. Half of forest fragments were lost over the resample period and six forests became smaller in size. Furthermore, majority of remnant forests changed markedly in forest structure and species composition. While monitoring of reserved forests is common (Taylor et al. 2008), we are not aware of long-term studies monitoring biodiversity and structure of private forest fragments in the tropics, especially in Africa. Human impact is likely to be the main driver behind the forest structure and composition changes observed. First, the forest fragments are not separated by large distances and are in the same climatic region, and thus variations in abiotic factors such as rainfall and temperature are too small to have a major effect on structure and species composition reported here. Second, the timescale involved (20 years) is too short, compared to the lifespan of trees, for compositional changes, especially for large size classes, to be explained by natural successional processes, although such processes may have some explanatory power for the new recruits (Connell and Green 2000). Most importantly variation across forests is consistently related to land use–related variables.

### Forest size, diameter-size class distribution, composition, and species richness change

In our fragments, the changes in forest size, forest structure, species richness, and composition as well as mean gap fraction are concomitant with disturbance level and land ownership. Reserved and research forests that were regularly patrolled and less disturbed retained similar species communities over the 20-year period. This is in agreement with studies that have showed high changes in forest gap fraction and size over time in unreserved forests compared to reserved forests and have been related to intense exploitation for timber and fuel wood, population growth, and increased urbanization (Puric-Mladenovic et al. 2000; Gaveau et al. 2009). Indeed the church forests without strict access control showed high levels of disturbance, significant reduction in stem densities, considerable changes in species communities, and had high gap fractions. This can be attributed to selective logging of small size classes as these are particularly easily accessed making them easy to remove without the owner being alerted. This is in line with an earlier study in the same forests that showed that small stems formed the highest proportion of tree stumps (Baranga et al. 2009). Such extractive activities affect the structural and forest floristic characteristics (Baranga et al. 2009), reduce regeneration (Veblen and Stewart 1980), change habitat heterogeneity, shift the competition balance among species, increase seedling and tree mortality, and alter the microclimate (Whitmore 1996; Garcia-Montiel and Scatena 1994; Boot and Gullison 1995; Denslow 1995). Across size classes, turnover is higher in the understorey than for the larger trees in church forests, whereas reserved and sacred forests had quite low turnover throughout all size classes. This compositional difference in the smaller size classes of the forest fragments may suggest potential for further taxonomic divergence in future canopy composition. The increase in small (<10 cm DBH) stems in one of the research forests (b) might be attributed to successful recruitment and low mortality in cocoa and tea experimental plots that were abandoned 50 years ago. Long-term studies in reserved forests have reported an increase in stem numbers through colonization until canopy space (basal area) is filled, and then decreases as larger trees subsume ever increasing proportions of the limited space (Sheil 2001). The dramatic increase in densities of stems 20–40 cm DBH in forests (i, j, and k) could be attributed to growth between the sampling periods, whereby the trees moves from one size class to another over the resample period.

Although we report dramatic changes in forest structure and composition, these changes are not reflected in the species richness of the majority of forest fragments. In fact, species richness increased over the resample period in four of the forest fragments, including several fragments that show strong indications of ongoing disturbance in terms of species composition and structure. Detailed analysis shows that in fragments where species richness increased, most of the species change can be attributed to early successional opportunistic species. This result agrees with other studies in tropical rain forests that have shown species richness peaked at intermediate disturbance levels particularly when disturbance intensity was estimated through the percentage of stems of strongly light-dependent species (Molino and Sabatier 2001). Our findings highlight the limitation of using species richness alone to assess forest integrity when biodiversity response to anthropogenic responses is varied and complex (see also Diffendorfer et al. 2007).

Eleven of the original fragments were lost over the resample period, and these have been converted into home gardens (1), human settlement (8), tourism development (1), and exotic tree plantations, mainly *Eucalyptus* and *Cupressus* for timber and firewood (1). This is congruent with East Africa's rapid expansion of privately owned exotic plantations estimated at 1.62% per annum over the last 10 years (FAO 2011). The presence of *S. spectabilis,* an invasive alien tree native to South and Central America (Irwin and Barneby 1982), in forest (j) is notable. This species colonizes gaps and more open forest, and would increase under any regime that increased disturbance (Sheil et al. 2000). Left uncontrolled, *S. spectabilis* has been shown to suppress recruitment of native species and thus reduce species richness (Wakibara and Mnaya 1980). To encourage regeneration of native species in invaded areas, its removal is recommended. *Artocarpus heterophyllus* which is naturalized in several forests has been noted to be invasive in Brazil (Zenni and Ziller 2011), but elsewhere it is not thought to be an aggressive invasive (Trevelyan et al. 2012).

Studies in South Asia and West and East Africa have demonstrated the importance of sacred groves in the conservation of biodiversity (Khumbongmayum et al. 2005; Sheridan 2009). The sacred forest in our study shows low taxonomic turnover over the resample period across size classes, and it contains large trees (>40 cm DBH) that have been preserved through strictly enforced beliefs of power and healing that can be attained through strict ritual performances inside these forests, taboos of bad luck and sickness that might arise from misuse of such forests, and customs supported by clan members from the Buganda kingdom (Gombya-Ssembajjwe 1997).

### Importance of private forests as habitats for endangered species

Some species listed by IUCN as “vulnerable” and “endangered” are recorded in the forest fragments, including *Lovoa swynnertonii* Bak.f., *Lovoa trichiloides* Harms*, Hallea stipulosa* (DC.) Leroy, *Entandrophragma angolense* (Welw.) C.DC*., Entandrophragma cylindricum* (Sprague) Sprague*, Entandrophragma utile* (Dawe & Sprague) Sprague*, Beilschmiedia* u*gandensis* Rendle, *Albizia ferruginea* (Guill. & Perr.) Benth.*, Milicia exelsa* (Welw.) C.C. Berg, and *Pouteria altissima* (A.Chev.) Baehni (IUCN 2011) (Appendix 1). Six species are important timber tree species and four occur as trees >10 cm DBH and are among the 20 most frequent species in 45% of the fragments sampled in 2010. This is in accord with studies in the Western Ghats that concluded that forest fragments supported over 70% of plant endemic species as well as substantial numbers of endemic animals (Muthuramkumar et al. 2006). Indeed, these forest fragments have been shown to harbor primate species particularly the red-tailed monkeys *Cercopithecus ascanius schmidti* (Baranga 2004b). Also significant numbers and changes have been recorded in moth species in these forests mainly driven by human disturbances (P. Akite, R. J. Telford, P. Waring, A. M. Akol, V. Vandvik, unpubl. ms.). The presence of such species supports that forest fragments can be important habitats for species of conservation interest, although more research is needed to establish if these private forests support viable (meta-) populations. Meta-population dynamics would be contingent on there being viable dispersal corridors linking the various fragments. In our study sites, the aerial photographs from 2010 show tree cover associated with private housing and mixed subsistence farming which could act as corridors for birds and primates, especially among close fragments such as d, e, g, and f but also roads, denser urban areas, and open fields which are likely to act as barriers for dispersal, especially for forests (i, h, j, and k). Forest connectivity (provided by forest corridors and matrix stepping stones) and landscape mosaic complexity have been found to be the main factors of spatial structure linked to the variations in tree and animal species (Metzger 1997; Prugh et al. 2008). The fragments themselves can be important stepping stones connecting protected areas within the region and at the same time we cannot understate their value for education and research.

## Conclusions

The majority of the Kampala area forests studied show high structural and compositional changes over a 20-year period. The observed changes are related to disturbance level, as well as ownership regimes. Despite increased degradation caused by logging and conversion, four of the forests show increasing species richness over the resampling period. Furthermore, the forest fragments harbor several near-threatened and vulnerable tree species as categorized by the IUCN (2010). Although at a local level these forests enhance biodiversity, at a regional level they are unlikely to be as important, and, furthermore, their importance must diminish as urbanization intensifies. More importantly, even with high forest loss, in the remnant forests, severe changes are occurring and the scenario is worse than forest loss alone indicates. A combination of management approaches needs to be implemented if the current deforestation and degradation is to halt. Particularly changing valuations of forests by owners, lack of incentives to maintain forest fragments in the landscape, and weak enforcement of ownership rights and regulations are immediate threats that need addressing. This could include encouraging proper management practices, identifying alternative sources of income such as eco-tourism, and engage in restoration activities particularly for forest that have been severely degraded such as the church forests in order to benefit from REDD+ carbon credits schemes. Promote nontimber forest products production within the forest area that compensates the full range of economic implications of avoiding deforestation. Even more important to ensure the future ecological and biodiversity value of forest fragments requires that: (1) further degradation is controlled; (2) reconnecting fragments through plantings to increase the overall size of the forested area as well as dispersal ability; and (3) degraded sites are enhanced by planting with native tree species. This might need a massive financial input, education, and outreach program requiring Uganda Government–private sector partnerships to execute.

## Appendix 1:

Tree species list DBH > 3.7 cm presence/absence of data for forest fragments described in Table 1 for the two sample periods.

**Table:**

	Forests codes																					
	
	a		b		c		d		e		f		g		h		i		j		k	
											
Species	2010	1990	2010	1990	2010	1990	2010	1990	2010	1990	2010	1990	2010	1990	2010	1990	2010	1990	2010	1990	2010	1990
*Acalypha neptunica* Muelll. Arg.	1	1	1				1	1		1		1					1	1		1		1
*Alangium chinensis* (Lour.) Rehder	1																1			1		
*Albizia gummifera* (J.L. Gmel.) C.A. Sm	1	1	1			1	1	1								1						
*Albizia zygia* (DC.) Macbr.			1			1	1			1	1	1		1			1	1				1
*Albizia coriaria* (Welw.ex) Oliv.														1					1	1		
*Albizia glaberrima* (Schumach. & Thonn.) Benth.						1																
*Albizia grandibracteata* Taub.												1										
*Alchornea cordifolia* Muell. Arg.						1										1		1				
*Alchornea floribunda* Muell. Arg.					1							1			1							
*Alchornea laxiflora* (Benth.) Pax & K. Hoffm.		1			1																1	
*Aningeria altissima* (A.Chev.) Aubrev. & Pellegr.	1	1	1			1	1			1		1			1	1	1	1				1
*Antiaris toxicaria* (Rumph.ex Pers.) Lesch.	1	1	1	1	1	1	1	1	1	1	1	1		1	1	1	1	1	1		1	1
*Antidesma membranaceum* Muell.		1	1		1	1									1	1			1			
*Aphania senegalensis* (Pior.) Radl.	1																					
*Argomuellera macrophylla* Pax																						1
*Artocarpus heterophyllus* Lam.					1	1							1		1		1	1	1			1
*Baikiaea insignis* Benth.	1																				1	
*Baphia capparidifolia* Bak.													1									
*Baphiopsis parviflora* Bak.						1																
*Barteria acuminata* E.G. Baker					1	1	1	1	1			1	1		1							
*Beilschmiedia ugandensis* Rendle^1^	1			1		1		1	1	1						1						
*Belanophora hypoglauca* (Welw. ex Hiern) A. Chev.	1	1														1						1
*Bersama abyssinica* Fres.		1				1		1						1				1				
*Blighia unijugata* Bak.	1	1	1	1	1	1	1	1	1	1		1	1	1	1	1	1	1		1		1
*Blighia welwitshii* (Hiern) Radlk.	1	1	1	1	1	1		1		1						1	1	1	1			
*Bridelia micrantha* (Hochst.) Baill.						1	1					1			1	1						1
*Bridelia scleroneura* Muell. Arg																			1			
*Canarium schweinfurthii* Engl.		1	1		1		1	1		1		1	1	1	1	1	1	1		1		
*Cedrela odorata* L.	1	1																				
*Celtis africana* Burm.f.	1	1	1				1	1		1					1			1		1		
*Celtis durandii* Engl.	1	1	1	1			1					1								1		
*Celtis gomphophylla* Engl.																	1					
*Celtis mildbraedii* Engl.	1	1	1		1													1			1	1
*Celtis zenkeri* Engl.	1	1																				
*Chaetacma aristata* Planch.	1	1	1			1	1	1	1	1		1			1		1	1	1	1	1	1
*Citrus limon* (L.) Burm.f.										1												
*Clausena anisata* (Willd) Benth.					1										1			1	1			1
*Cnestis.ugandensis* Schellenb	1																					
*Coffea canephora* Froehner			1												1	1		1	1		1	
*Coffea. eugenioides* S. Moore								1	1							1						
*Coffea robusta* Pierre			1																1	1		
*Combretum molle* G.Don																			1			
*Combretum racemosum* P. Beauv.						1																
*Connarus longistipitatus* Gilg										1		1										
*Craterispermum schweinfurthii* Hiern	1				1	1				1				1		1						1
*Croton macrostachyus* Hochst. ex A. Rich.					1												1			1		
*Croton sylvaticus* Krauss																	1					
*Deinbollia kilimandscharica* Taub.			1		1										1		1		1			
*Deinbollia fulvo-tomentella* Bak.f.	1																					
*Dictyandra arborescens* Welw.ex Benth. & Hook. f.	1	1				1	1	1		1		1				1						1
*Diospyros abyssinica* (Hiern) F White			1																1			
*Dovyalis macrocalyx* (Oliv.) Warb.																						1
*Dracaena fragrans* (L.) Ker-Gawl.		1																				
*Drypetes ugandensis* (Rendle) Hutch.	1				1	1										1						
*Ekebergia capensis* Sparrm.					1													1				
*Englerophytum oblanceolatum* (S. Moore) T.D. Penn.	1																					
*Entandrophragma angolense* (Welw.) C.DC.^1^	1				1	1	1								1							
*Entandrophragma cylindricum* (Sprague) Sprague^1^	1																					
*Entandrophragma utile* (Dawe & Sprague) Sprague^1^	1	1		1		1						1				1						
*Erythrina Erythrococca* Bak.	1		1	1											1		1	1				
*Erythrococca artrovirens* Pax Prain							1	1		1						1						
*Erythrophleum suaveolens* (Guill. & Perr.) Brenan					1	1	1	1		1	1	1			1	1						1
*Eucalyptus grandis* W. Hill													1									
*Eugenia capensis* (Eckl. & Zeyh.) Sond.								1	1				1			1					1	1
*Euphorbia teke* Scweinf. ex Pax.	1	1	1												1							
*Fagaropsis angolensis* Engl. Dale	1											1								1		
*Ficus asperifolia* (Miq.) Miq.			1						1				1				1					
*Ficus polita* Vahl																	1					
*Ficus capensis* Thunb.														1						1		
*Ficus exasperata* Vahl	1	1	1												1	1				1		
*Ficus mucoso* Welw.	1	1	1												1		1	1				
*Ficus natalensis* Hochst.																				1		
*Ficus sur* Forssk.							1								1		1					
*Ficus sycomorus* L.	1																					
*Ficus variifolia* Warb.															1							
*Funtumia africana* (Benth.) Stapf.		1		1																		
*Funtumia elastica* (P. Preuss) Stapf	1	1	1	1			1	1	1	1	1	1	1		1	1		1	1			1
*Garcinia buchananii* Bak.												1				1					1	1
*Hallea stipulosa* (DC.) Leroy^1^	1																					
*Harungana madagascariens* Poir.							1							1		1	1		1	1		1
*Heisteria parvifolia* Smith					1																	
*Hugonia platysepala* Welw.									1													
*Kigelia africana* (Lam.) Benth.		1																				
*Lannea welwitschii* (Hiern) Engl.	1	1						1							1		1					
*Lepidotrichilia volkensii* (Gurke) Leroy															1							
*Leptonychia mildbraedii* Engl.	1																					
*Lindackeria bukobensis* Gilg.																				1		
*Lovoa swynnertonii* Bak.f.^1^	1	1																				
*Lovoa trichilioides* Harms^1^	1	1	1	1	1	1	1	1	1	1	1	1			1	1	1	1		1	1	1
*Lychnodiscus cerospermus* Radlk.		1																				
*Macaranga angolensis (*Muell. Arg.)			1		1		1		1						1							
*Macaranga holstii* Engl.	1							1		1						1						
Macaranga monandra Muell. Arg.	1		1		1		1		1								1					
*Macaranga pynaertii* De Wild.	1		1			1		1	1	1		1				1		1		1		1
*Macaranga schweinfurthii* Pax	1		1	1												1						
*Macaranga spinosa* Mull. Arg.							1								1	1				1		
*Maerua duchesnei* (De Wild.) F. White							1															
*Maesa welwitschii* Gilg			1												1					1		
*Maesopsis eminii* Engl.	1		1	1	1	1	1	1	1	1	1	1	1	1	1	1	1	1	1	1	1	1
*Mallotus oppositifolius* (Geisel.) Mull. Arg	1																					
*Mangifera indica* L.					1					1			1		1							
*Manihot glaziovii* Mull. Arg.								1														
*Manihot* spp.							1															
*Manilkara butugi* Chiov.	1	1	1	1	1	1	1	1		1		1	1		1			1		1		
*Manilkara dawei* (Stapf) Chiov.		1				1										1						1
*Margaritaria discoidea* (Baillon) Webster (trop. Afr.)	1			1	1	1	1		1	1		1		1	1	1	1	1		1		
*Markhamia lutea* (Bak.) Sprague			1																			
*Memecylon jasminoides* Gilg.																			1		1	
*Milicia exelsa* (Welw.) C.C. Berg	1		1				1		1		1				1		1		1			
*Mimusops bagshawei* S. Moore	1	1				1	1			1		1		1			1					
*Mitrogyna rubrostipulata* (K. Schum.) Havil.			1																			
*Monodora myristica* (Gaertn.) Dunal												1			1	1						
*Montandra guinense* (Thonn)							1															
*Morinda lucida* Benth.	1													1			1					
*Morus mesozygia* Stapf	1	1																				
*Musanga leo-errerae* Hauman & J. Leonard			1				1								1	1	1	1				
*Myrianthus arboreus* Beauv.							1					1										
*Myrianthus holstii* Engl.			1	1			1	1		1		1			1	1	1					
*Ochna afzelii* R. Br.ex.Oliv.					1	1													1		1	1
*Ochna holstii* Engl.					1																1	
*Olax gambecola* Baill.						1																
*Olea capensis* L.	1																					
*Oxyanthus speciosus* DC.	1		1	1	1	1	1	1	1	1		1			1	1	1			1		
*Oxyanthus ugandensis* Bridson		1		1			1															
*Oxyanthus unilocularis* Hiern	1											1					1					
*Pachystela brevipes* (Bak.) Engl.		1				1	1															
*Pancovia pedicellaris* Radlk. & Gilg.																1						
*Pancovia turbinata* Radlk.						1																
*Parkia filicoidea* Welw. ex. Oliv.	1		1	1	1	1	1			1					1	1		1				
*Paropsia guineensis* Oliv.	1	1			1	1		1								1	1	1				
*Pavetta moludensis* K.Krause		1	1					1		1		1										
*Pavetta oliveriana* Hiern		1																				
*Pinus patula* Schltdl. & Cham.											1											
*Piptadeniastrum africanum* (Hook.f.) Brenan	1	1	1	1	1	1		1	1	1	1	1			1	1	1		1	1	1	1
*Pittosporum mannii* Hook.f.						1		1				1		1	1	1		1	1	1	1	1
*Polyscias fulva* (Hiern) Harms	1				1	1	1	1	1						1		1		1			
*Pouteria altissima* (A.Chev.) Baehni^1^							1															
*Pseudospondias microcarpa* (A. Rich.) Engl.	1	1	1	1	1	1	1	1	1	1	1	1	1	1	1	1	1	1	1	1	1	
*Psidium guajava* L.													1	1						1		
*Psydrax parviflora* (Afzel.) Bridson							1		1												1	
*Pycnanthus angolensis* (Welw.) Warb.	1	1	1		1	1	1	1	1	1	1	1	1	1	1	1	1	1		1	1	
*Rhus natelensis* Bernh. ex Krauss																			1			
*Ricinondendron heudelotii* (Baill.) Heckel	1																					
*Rothmannia longiflora* Sansb.	1				1																	
*Rothmannia urcelliformis* (Hiern) Bullock ex Robyns	1		1				1	1							1			1				
*Rouvolfia vomitoria* Afzel.															1							
*Rytigynia bagshawei* (S. Moore) Robyns		1																				
*Rytigynia beniniensis* (De Wild.) Robyns								1	1	1						1	1			1		
*Sapium ellipticum* (Hochst.ex Krauss) Pax			1			1		1	1	1		1		1	1	1	1	1	1	1	1	1
*Scolopia rhamnophylla* Gilg								1	1													
*Senna spectabilis* DC.																			1			
*Solunum mauritaiana* Scop.			1			1						1		1								
*Spondianthus preusii* Engl.	1				1		1	1		1		1	1	1	1	1				1		
*Spondianthus ugandensis* Hutch.							1															
*Staudtia kamerunensis* Warb.	1	1			1	1	1	1		1					1	1					1	
*Sterculia dawei* Sprague	1						1								1	1	1		1	1		
*Symphonia globulifera* L.f.					1	1																
*Synsepalum brevipes* (Baker) T.D. Penn.					1												1					
*Syzygium guineense* (Willd.) DC.	1	1	1	1						1				1		1			1	1		1
*Tabernaemontana holstii* K. Schum	1	1	1	1	1	1	1	1	1	1		1			1	1	1					
*Tarenna pavettoides* (Harv.) Sim			1						1													
*Terminalia ivorensis* A. Chev.	1														1							
*Tetraplaura. tetraptera* (Schumach. & Thonn.) Taub.	1																					
*Tetrorchidium didymostemon* (Baill.) Pax & K. Hoffm.					1	1	1	1	1	1		1		1	1		1	1				
*Thecacoris lucida* (Pax) Hutch.																					1	
*Theobroma cacao* L.			1	1																		
*Treculia africana* Decne	1	1	1		1	1		1						1						1		
*Tremma orientalis* (L.) Blume	1							1						1		1		1				1
*Trichilia dregeana* Sond.	1	1	1	1	1	1	1	1		1			1	1	1	1	1	1		1		1
*Trichilia martineaui* Aubrev. & Pellegr.	1				1																	
*Trichilia prieuriana* A. Juss.	1	1	1	1	1	1		1		1		1				1	1	1	1	1	1	1
*Trichilia rubescens* Oliv.	1	1	1		1	1	1		1							1			1			
*Trilepisium madagascariense* DC.	1	1	1	1	1	1	1	1	1	1		1			1	1	1	1		1	1	1
*Uvaria angolensis* Oliv.						1																
*Vangueria apiculata* K. Schum.		1						1								1			1	1		1
*Vespris nobilis* Engl.	1	1	1	1	1	1	1	1	1	1		1				1	1	1	1	1		
*Vitex ferruginea* Schumach. & Thonn.																			1		1	1
*Voacanga africana* K. Schum	1																					
*Warneckea jasminoides* Gilg	1				1		1															
*Xylopia staudtii* (Dunal) A.Rich.					1																	1
*Xylopia. rubescens* Oliv.			1																			
*Xymalos. manii* (Harv.)	1															1						
*Xymalos. monospora*. (Harv.) Baill.	1														1							
*Zanha. golungensis* Hiem	1	1	1		1	1	1								1							
*Zanthoxylum rubescens* Hook.f.							1															
*Zanthoxylum chalibeaum* Engl.										1						1			1			
*Zanthoxylum. gilletii* (De Wild.) P.G. Waterman					1	1																
*Zanthoxylum. leprieurii* Guill. & Perr.	1				1		1									1						

See Table 1 for forest codes. ^1^IUCN red-list species.
